# A Five-Year Followup of Human Seminal Plasma Allergy in an 18-Year-Old Woman

**DOI:** 10.1155/2012/257246

**Published:** 2012-05-08

**Authors:** Ole D. Wolthers

**Affiliations:** Asthma and Allergy Clinic, Children's Clinic Randers, 8900 Randers, Denmark

## Abstract

Case reports of women with the rare condition of human seminal plasma allergy have indicated that the condition may be associated with life-threatening anaphylactic reactions in relation to coitus. Few observations, if any, of long-term outcome of the condition are available. The aim of this paper was to present a case diagnosed in an 18-year-old girl who presented with generalized urticaria, nasal congestion and secretion, conjunctivitis, and periorbital and labial oedema 6–8 hours after coitus. During five years of followup the condition improved clinically significantly. Due to intimacy concerns and the low prevalence of the condition robust long term data on the natural course of the condition are difficult to obtain. The present case suggests that in some patients the condition may improve over time.

## 1. Introduction

Human seminal plasma allergy is an anaphylaxis caused by immediate hypersensitivity to human seminal plasma. Around 80 cases have been published since 1958, none of which have been in adolescents [1]. The condition is considered to be underreported. Management of the condition includes abstinence, condom usage, pharmacologic symptomatic treatment, subcutaneous immunotherapy, and local intravaginal desensitization with human seminal plasma. Few observations, if any, of long-term outcome of the condition are available. We report a 5-year followup of a case of human seminal plasma allergy in a young woman which suggests that long-term spontaneous improvement may occur.

## 2. Case

An 18-year-old woman with a history of eczema during childhood presented with complaints of episodes of generalized urticaria, nasal congestion and secretion, conjunctivitis, and periorbital and labial oedema 6–8 hours after coitus. There was no vaginal itching or burning.

 Total IgE was slightly elevated, 350 (0–250) KIU/L. The Phadia Immunocap and the Multiple Allergen Simultaneous Test-chemiluminescent Assay (MAST CLA) test panels were negative [2]. Histamine release tests for latex, the partners saliva, sweat and semen were performed. The histamine release test procedure includes histamine release from peripheral whole-blood basophils, in a glass fiber prepared microtiter plate, by challenging 25 *μ*L heparinised whole blood with 25 *μ*L buffered allergen dilutions in various concentrations for 60 minutes at 37°C. The assay is calibrated by a two-point standard curve and a positive cell control (anti-IgE) [3]. The test results are given as negative or positive. Histamine release tests for latex and for the partners saliva and sweat were negative, whereas the test was positive for the partners semen. The partners sweat, saliva, and semen were also tested in blood from a healthy control person, and these tests were all negative. Furthermore, a skin prick test was performed in the patient finding a positive reaction with a diameter of 12 (positive control 5) mm for the partners semen. Skin prick tests with the partners semen were also performed in two healthy women finding negative results.

 During the first 6 months after a diagnosis of anaphylaxis to human seminal plasma was established, condom usage was not sufficient for prophylaxis. Even close body contact such as kissing and hugging would cause acute urticaria, sneezing, and conjunctivitis despite daily intake of oral antihistamines, however, life-threatening anaphylactic reactions did not occur. Sexual intercourse was only possible on prednisolone 25 mg/day; so, the couple opted for abstinence and slept in separate rooms during most of that period. Between 6 and 12 months after the diagnosis was established, gradually, body contact became possible, and some months later with the use of a condom and on premedication with oral antihistamines coitus was possible without associated symptoms. During the second and third years after diagnosis the patient would still use oral antihistamines prior to intercourse several times per week; however, the condition was challenged on a regular basis by the couple showing milder and milder symptoms. Five years after the diagnosis incidental episodes of provocation caused by condom defects and intended challenges without condom were not associated with significant symptoms. A skin prick test in the patient for the partners semen at that time found a positive reaction of a diameter of 10 (positive control 7) mm. The patient did not report any other sexual partners.

## 3. Discussion

Hypersensitivity to human seminal plasma is defined as a spectrum of systemic and/or localized symptoms after exposure to specific protein components in seminal plasma [1]. The diagnosis is based on the medical history in combination with avoidance of symptoms by correct condom usage and conventional tests of IgE-mediated allergy. Depending on history differential diagnoses of hypersensitivity to latex, spermicidal agents, lubricants, seminal-mediated food challenges, and microbial causes of vaginal burning and itching may need to be considered [1]. Absence of symptoms with condom use is considered the gold standard of diagnosis; however, as illustrated by the present case condom usage may not be efficient prophylaxis in all cases [4]. The prevalence of the condition in the unselected population remains unknown as do implications for sexual health and fertility. The major antigen is believed to be prostate-specific antigen, but other proteins may be involved [5]. The condition is generally believed to be species-specific [1]. Women who develop systemic symptoms are more frequently atopic; however, significant risk factors have not been identified. As evidenced by the present case the condition may present without detectable symptoms of an immediate type 1 allergy reaction such as vaginal burning or itching. Published cases so far have been in the 20- to 50-year age group. Most of them were diagnosed because the women were admitted to hospital with life-threatening anaphylaxis or because they were seeking medical advice with a wish for pregnancy. It is generally believed that the condition is significantly underreported since milder cases do not seek help because of intimacy concerns. That may also explain why cases in adolescents have not been reported until now. A further explanation may be that in some patients the condition may improve over time as illustrated by the present case so that the condition may not significantly affect long-term sexuality or fertility. The present observation of clinical improvement was not supported by any measures of immunomodulation such as a shift from a Th2 to a Th1 response, and, indeed, no such data are available in the literature. Clinical tolerance may be medically induced over few months by subcutaneous immunotherapy to relevant fractionated seminal plasma proteins and by instillation of intravaginal graded challenge using dilutions of whole seminal fluid from the woman's sexual partner [5, 6]. As suggested by the present report perhaps in some women such procedures may be avoided unless the patient has an urgent wish for pregnancy. Having said that, one needs to acknowledge that the possibility of spontaneous improvement in the unselected population remains unknown. Due to intimacy concerns and the low prevalence of the condition robust data are extremely difficult to obtain.
